# Differential Wnt/β-catenin signaling via TCF7L2/LEF1 binding specificity shapes cellular and tumor phenotypes

**DOI:** 10.1073/pnas.2528450123

**Published:** 2026-06-10

**Authors:** Thomas A. Kluiver, Anna Nordin, Yuyan Lu, Xuan Guo, Stephanie A. Schubert, Darien Yeung, Arif Ibrahim Ardisasmita, Wessel Terpstra, Chang Zhang, Xiaochen Duan, Rishi Savur, Marius C. van den Heuvel, Vincent E. de Meijer, Ruben H. de Kleine, Kathelijne Kraal, Ronald R. de Krijger, József Zsiros, Claudio Cantù, Weng Chuan Peng

**Affiliations:** ^a^ Princess Máxima Center for Pediatric Oncology, Utrecht 3584 CH, The Netherlands; ^b^ Wallenberg Centre for Molecular Medicine, Linköping University, Linköping SE-581 83, Sweden; ^c^ Department of Biomedical and Clinical Sciences, Division of Molecular Medicine and Virology, Faculty of Medicine and Health Sciences, Linköping University, Linköping SE-581 83, Sweden; ^d^ Science for Life Laboratory, Linköping University, Linköping SE-581 83, Sweden; ^e^ Department of Oncology, Zhongshan Hospital of Xiamen University, School of Medicine, Xiamen University, Xiamen 361004, China; ^f^ Department of Surgery, Section of Hepatobiliary Surgery and Liver Transplantation, University of Groningen, University Medical Center Groningen, Groningen 9713 GZ, The Netherlands

**Keywords:** Wnt signaling, β-catenin, TCF/LEF, cancer, novel motif

## Abstract

The mechanisms by which Wnt/β-catenin signaling regulates gene expression in a tissue- and context-specific manner remain poorly understood, limiting our ability to target the aberrant cell growth typical of many Wnt-driven cancers. Here, we focus on malignant liver tumors driven by activating *CTNNB1* (β-catenin) mutations that nevertheless display distinct phenotypic states and Wnt outputs. By profiling patient-derived organoids via single-cell transcriptomics and chromatin dynamics, we identify subtype-specific transcriptional and epigenetic profiles. Using CUT&RUN, we show that β-catenin engages distinct genomic regions, dictated by differential association with TCF/LEF family transcription factors. Specifically, we define a sequence-specific regulatory element engaged by β-catenin only upon interaction with TCF7L2, revealing that partner choice, independent of *CTNNB1* mutational status, ultimately determines cell fate. Our findings, validated across multiple tumor models and patient tissues, offer a framework for understanding how differential β-catenin-TCF/LEF interaction orchestrates context-specific Wnt signaling outcomes.

## Significance

Wnt/β-catenin signaling is crucial for development and cancer, yet how it drives different gene programs across tissues is unclear. Using patient-derived liver tumor organoids, we show that β-catenin’s transcriptional output depends on its binding partner: LEF1 or TCF7L2. These factors guide β-catenin to distinct genomic regions, activating either stemness or differentiation genes. We identify a novel helper motif that directs β-catenin–TCF7L2 binding and target selection. By linking partner choice and motif specificity to context-dependent gene regulation, our work provides a unifying mechanism explaining how Wnt/β-catenin signaling produces diverse cellular outcomes.

Wnt/β-catenin signaling is a cell communication mechanism fundamental for regulating cellular proliferation and fate specification during embryogenesis and tissue homeostasis (1, 2). Its mechanism of action centers on Wnt ligand-dependent stabilization of cytosolic β-catenin. Once stabilized, β-catenin travels to the nucleus where it forms a complex with a member of the T cell factor/lymphoid enhancer factor (TCF/LEF) transcription factor family to regulate Wnt target gene expression. TCF/LEF, by DNA motif recognition, determine the genomic Wnt responsive elements (WREs) (3). However, how β-catenin regulates different genes depending on the cell type remains an outstanding question in the field.

Dysregulation of Wnt/β-catenin signaling causes developmental abnormalities and many cancers (4, 5). Among cancers, hepatoblastoma offers a particularly tractable model to interrogate this pathway in cancer. It is a developmental tumor that is typically driven by activating mutations in *CTNNB1* (encoding β-catenin), with less frequent alterations in other Wnt pathway genes, and otherwise exhibits a very low mutational burden (6, 7). This relatively “clean” genetic background and well-defined phenotypic subtypes provide a powerful framework to dissect how the Wnt/β-catenin pathway regulates lineage programs and malignant behavior.

Two main epithelial cell types are commonly observed in hepatoblastoma: the less differentiated “embryonal” histologic subtype (E), characterized by high expression of classical Wnt target genes and associated with worse prognosis (8, 9), and the more differentiated “fetal” histologic subtype (F), which resembles the fetal liver developmental stage (10). Recent technologies confirmed this classification on a molecular level (6, 11–21). However, it remains unclear how common genetic driver mutations that stably activate β-catenin can lead to different outcomes. This is particularly puzzling given that E and F subtypes often coexist within the same tumor (10). Thus, liver tumors of developmental origin offer a unique and naturally occurring context to study how overactivation of Wnt/β-catenin signaling could drive distinct cellular outcomes.

Here, we use patient-derived tumor organoids (PDOs) representing the two subtypes (20). By integrating single-cell RNA sequencing (scRNA-seq), single-cell ATAC-seq, and CUT&RUN targeting β-catenin and TCF/LEF, we find that β-catenin physically associates with different genomic elements between E and F cells. We find that the chromatin state and other lineage-specific transcription factors only partially explain this differential β-catenin activity. More surprisingly, we find that the determinant of β-catenin activity is attributed to the distinct DNA-binding activity of the two main TCF/LEF transcription factors expressed, LEF1 and TCF7L2. LEF1, in the E subtype, is associated with the classic consensus sequence present in well-characterized WREs, while TCF7L2 is also associated with a different DNA motif and drives β-catenin to regulate liver differentiation and metabolism genes. This consensus-mediated genomic association occurs across cell types and tumor models, revealing this as a universal mechanism of Wnt/β-catenin signaling regulation.

Collectively, our study leverages an integrative multiomics approach in patient-derived organoids to elucidate fundamental aspects of Wnt signaling-driven transcriptional regulation. These insights expand our understanding of Wnt signaling dynamics and establish a robust experimental platform for the investigation of cellular subtype-specific regulatory mechanisms in the context of cancer and developmental biology.

## Results

### Divergent Wnt Gene Expression Programs Despite Constitutive Activation of β-Catenin.

**Fig. 1.**
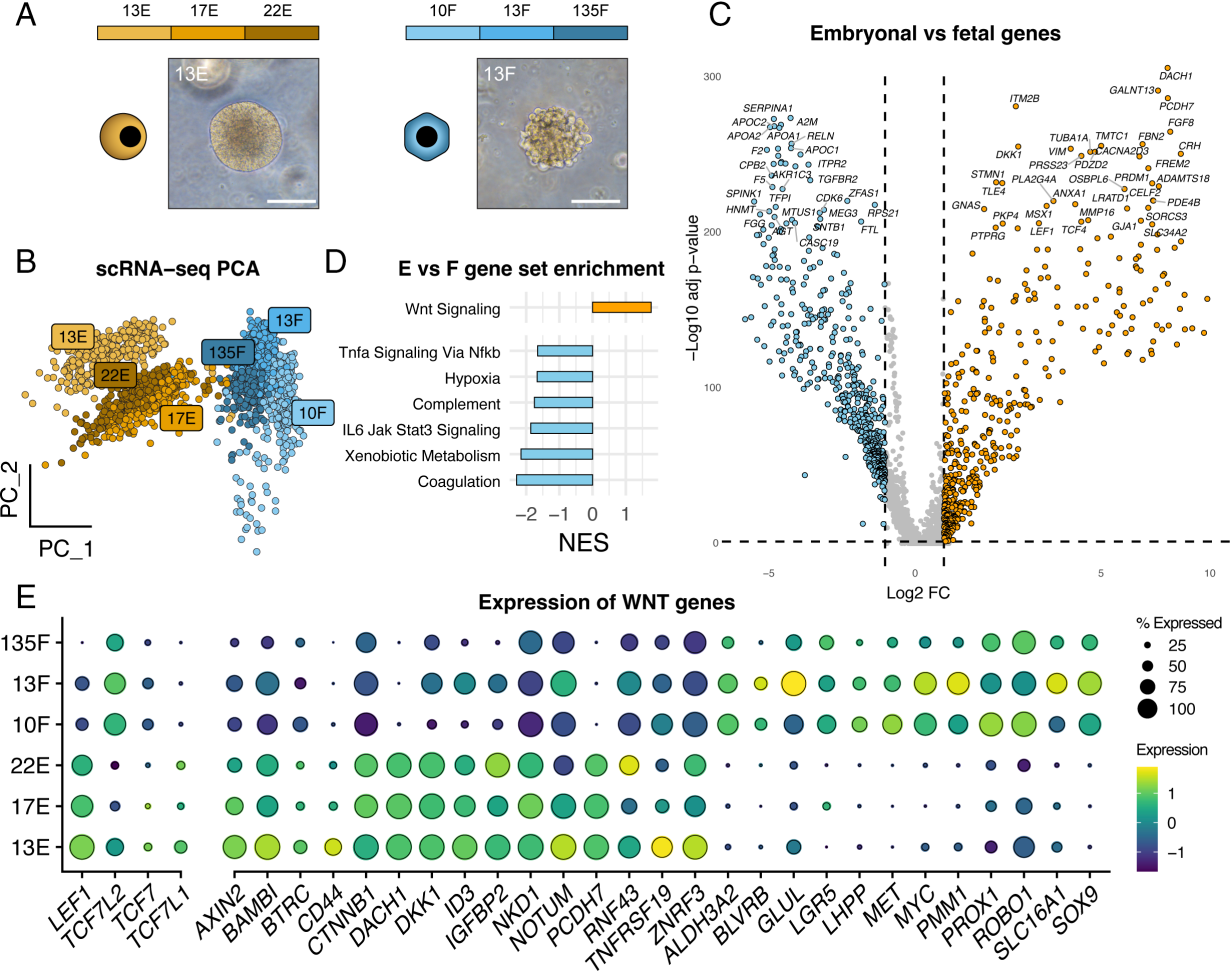
Single-cell transcriptome analysis of PDOs (*A*) Overview of the six organoid models used in this study, with phase-contrast pictures of representative 13E and 13F organoids. (Scale bar, 100 µm.) (*B*) scRNA-seq PCA embedding splits embryonal and fetal tumor cells. (*C*) Volcano plot showing differentially expressed genes between the two subtypes. (*D*) Gene set enrichment plot showing enrichment for Hallmark gene sets in both subtypes. NES: normalized enrichment score. (*E*) Dot plot showing expression of Wnt targets and related genes in embryonal (E) and fetal (F) models.

We employed six unique patient-derived organoid models, representative of E (13E, 17E, and 22E) and F subtypes (10F, 13F, and 135F) (Fig. 1*A*) (20). Among these, 13E and 13F were derived from the same patient (Fig. 1*A*). Single-cell RNA sequencing (scRNA-seq) performed on these organoids (20) confirmed a PCA-based separation of the two groups, and presence of distinct cellular features (Fig. 1 *B*–*D* and *SI Appendix*, Fig. S1 *A* and *B*). The F subtype expressed high levels of differentiated hepatic markers (*SERPINA1*, *APOA1*, and *ALB*) and showed enrichment for gene sets involved in hepatic functions, including xenobiotic metabolism and coagulation (Fig. 1 *C* and *D*). Conversely, the E subtype expressed high levels of genes associated with stemness (*MSX1*, *PRDM1*, and *STMN1*) and epithelial plasticity (*VIM*, *ANXA1*, *MMP16*, and *GJA1*) (Fig. 1*C*). This was accompanied by strong enrichment of the Wnt signaling gene set (Fig. 1*D*). The differential expression of Wnt/β-catenin related genes was notable (Fig. 1*E*). The E subtype displayed elevated expression of canonical Wnt-associated genes, including *NKD1*, *NOTUM*, *DKK1*, *DACH1*, *MSX1*, *VIM*, and *FGF8*. In contrast, the F subtype showed lower expression of these genes but higher expression of Wnt-regulated metabolic genes (*GLUL* and *OAT*) and additional Wnt targets such as *LGR5*, *ROBO1*, *MYC*, *PROX1,* and *SOX9* (Fig. 1*E*) (22). These data suggest the presence of a shared Wnt program across both subtypes, as well as subtype-specific enrichment of Wnt target genes.

Next, we examined the expression of TCF/LEF transcription factors, the main DNA binding partners of nuclear β-catenin. Among these, *TCF7L2*, a Wnt effector broadly expressed in the liver and known to regulate hepatic metabolic programs (22), was detected in both E and F subtypes, with higher expression in the F subtype (Fig. 1*E* and *SI Appendix*, Fig. S1*C*). Isoform analysis showed expression of the E and M variants of *TCF7L2* (i.e., with and without the DNA-binding C-clamp domain, respectively), whereas the S isoform (with truncated C-clamp) was not detected (*SI Appendix*, Fig. S1 *D*–*G*) (23, 24). In contrast, *LEF1*, typically associated with a progenitor phenotype and stemness in development and cancer (25), is specifically upregulated in the E organoids (Fig. 1*E* and *SI Appendix*, Fig. S1*C*). Notably, LEF1 is generally absent in mature hepatocytes, and its expression is consistent with a less differentiated, more progenitor-like identity in E organoids. The expression of *TCF7* and *TCF7L1* was low in both subtypes, indicating a limited role for these transcription factors in hepatoblastoma (Fig. 1*E*). Next, we performed siRNA-mediated knockdown of *TCF7L2* and *LEF1* in F (13F) and E (13E) organoid lines. At day 7, siTCF7L2 and siTCF7L2-LEF1 double knockdown significantly reduced cell viability in both subtypes while siLEF1 alone, surprisingly, showed a modest increase (13F) or no significant effect (13E) in cell viability (*SI Appendix*, Fig. S1*H*). We also performed RT-qPCR analysis of canonical Wnt targets, using samples harvested at day 3, and generally observed a downregulation trend in both subtypes (*SI Appendix*, Fig. S1*H*). The 13E organoid line was affected by siLEF1 and/or siTCF7L2 while the 13F line was more selectively affected by siTCF7L2. We also examined the expression of E-only targets such as *ITM2B* and *CRH*, which decreased upon *LEF1* knockdown in 13E. Next, we examined hepatic functional genes such as *FABP1* and *PAH*, and we observed upregulation upon *TCF7L2* knockdown. This unexpected increase suggests that TCF7L2 may repress hepatic differentiation markers likely through Groucho/TLE corepressors, consistent with previous observations that TCF7L2 knockdown can increase differentiation genes (22, 26–29).

### Subtype-Specific Chromatin Accessibility and Motif Enrichment.

**Fig. 2.**
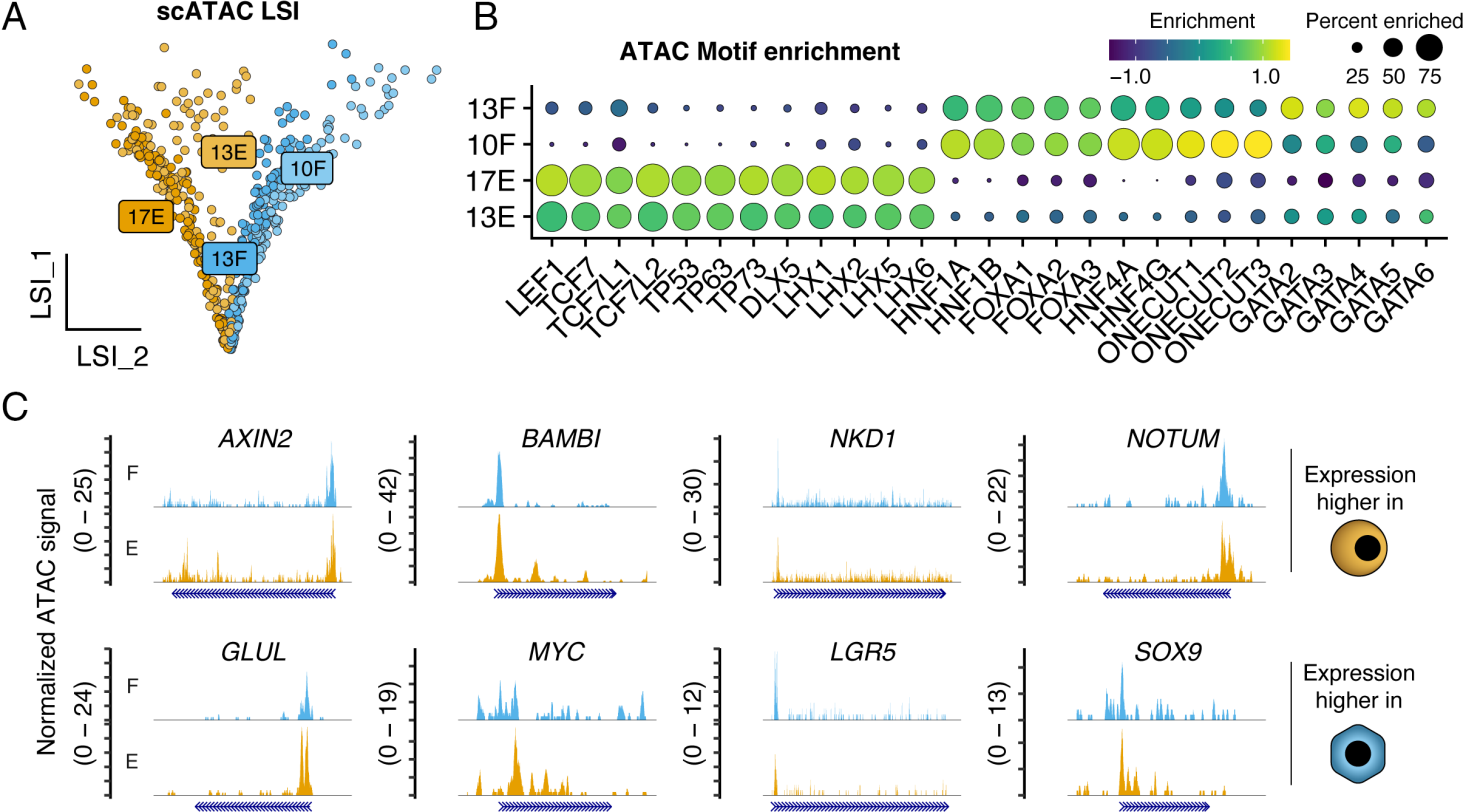
Chromatin accessibility analysis of PDOs. (*A*) scRNA/ATAC-seq LSI embedding of embryonal and fetal tumor cells. (*B*) Dot plot showing ATAC motif enrichment of selected TF families. (*C*) Coverage plots showing ATAC signal at promoter regions of Wnt targets that were overexpressed in either E or F organoids.

To investigate whether the differential Wnt/β-catenin responses observed in E vs. F organoids are caused by subtype-specific epigenetic features, we utilized our single-nucleus chromatin accessibility profiling parallel to transcriptomics (scATAC/RNA-seq) dataset featuring four organoid models (13E, 17E, 10F, and 13F). Dimensionality reduction using ATAC-based latent semantic indexing (LSI) separated the organoids by subtype, consistent with PCA and UMAP analyses (Fig. 2*A* and *SI Appendix*, Fig. S2*A*), indicating that the two subtypes have different chromatin states. Motif enrichment analysis performed across the sequence underlying the ATAC-seq peaks revealed subtype-specific signatures: E organoids were enriched for motifs of TCF/LEF, p53, and DLX/LHX transcription factors, while F organoids showed motifs of hepatic regulators, such as GATA, FOXA, ONECUT, HNF1A/B, and HNF4A/G (Fig. 2*B* and *SI Appendix*, Fig. S2*B*). Thus, the heterogeneity in chromatin accessibility mirrors that observed via scRNA-seq, indicating distinct transcriptional networks.

We then examined the chromatin accessibility at promoters of Wnt targets enriched in each subtype, including *AXIN2*, *BAMBI*, *NKD1,* and *NOTUM* for E organoids and *GLUL*, *MYC*, *LGR5,* and *SOX9* for F organoids (Figs. 1*E* and 2*C*). Surprisingly, promoter accessibility was comparable between subtypes (Fig. 2*C*). Additionally, the F subtype showed increased accessibility at promoters of hepatic genes such as *ASGR1*, *APOA2*, *APOB,* and *TF* (*SI Appendix*, Fig. S2*C*). Overall, while broader chromatin landscapes and motif enrichment differ between subtypes, promoter accessibility at common Wnt targets appeared comparable.

### β-Catenin Displays Subtype-Specific Genome-Wide Binding.

**Fig. 3.**
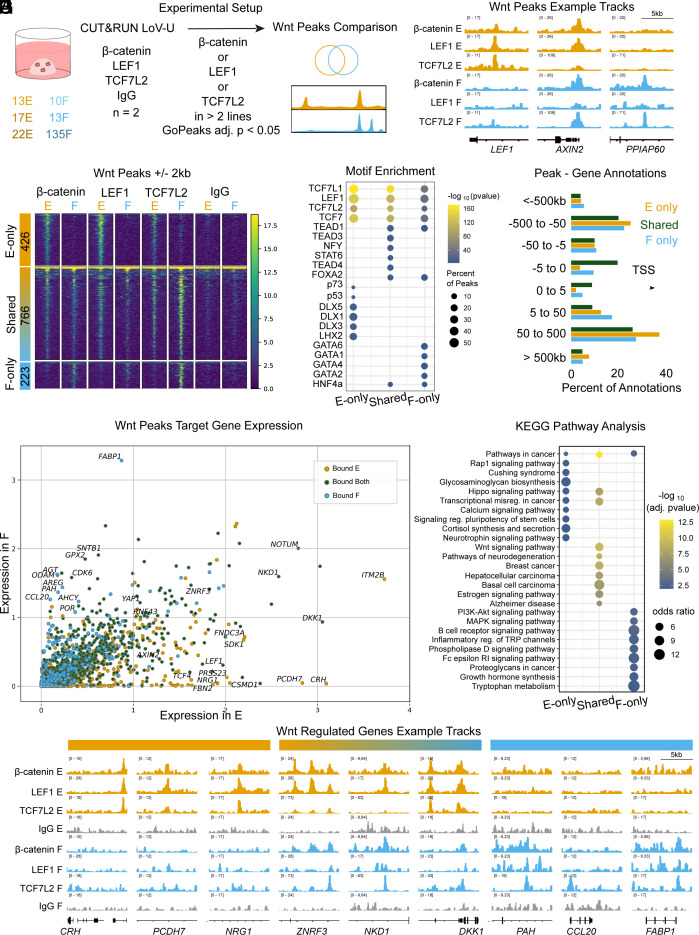
CUT&RUN analysis reveals subtype-specific Wnt/β-catenin binding activity. (*A*) Experimental design overview and the definition of Wnt peak sets. (*B*) Example genome browser visualization of Wnt peaks specific to embryonal lines (*LEF1,* *Left*), common to both (*AXIN2*, *Center*) and specific to fetal lines (*PPIAP60*, *Right*). For genome browser visualization, normalized bigwigs from all lines from each subtype and target were averaged. (*C*) Heatmaps of signal centered within peak regions and showing +/− 2 kb for all CUT&RUN targets over E-only peaks (*Top*, 426), shared (*Middle*, 766), and F-only peaks (*Bottom*, 223). Heatmaps are based on the averaged bigwigs. (*D*) Motif enrichment dot plot of HOMER identified known motifs in E-only, shared and F-only Wnt peak regions, showing high enrichment of TCF/LEF consensus sequences as well as subtype-specific motifs. Top 10 motifs are shown, cutoff q < 0.05, dot color represents significance and dot size percentage of peaks. (*E*) Peak-gene annotation frequencies according to GREAT analysis, showing a higher instance of promoter occupancy in shared Wnt peaks. (*F*) Graph showing expression in *E* (x-axis) vs. expression in *F* (y-axis) for all annotated Wnt target genes. Dots are colored based on bound status. (*G*) KEGG pathway enrichment dot plot based on expressed target genes bound only in *E*, bound in both, or only in *F*. Top 10 results are shown, cutoff adj. *P* < 0.05. Dot color represents significance and size represents odds ratio. KEGG analysis was done with all expressed genes as a background set. (*H*) Additional genomic loci of E-only targets (*Left*), shared Wnt target genes (*Center*) and F-only targets (*Right*).

We next assessed whether differential recruitment of β-catenin and its TCF/LEF partners to DNA might explain the divergent Wnt signaling outcomes. We mapped genome-wide binding of β-catenin and TCF/LEF family members with CUT&RUN-LoV-U (30) on the E and F organoids (Fig. 3*A*, PCA in *SI Appendix*, Fig. S3*A*). LEF1 and TCF7L2 were the only TCF/LEF factors yielding reliable signal due to their higher expression pattern. After quality control we merged replicates, called peaks using GoPeaks (adj. *P* < 0.05) against the matching IgG negative controls, and compiled a set of “Wnt peaks” for each subtype: loci bound by β-catenin together with LEF1 or TCF7L2 in at least two of the three organoid lines (Fig. 3*A*, *SI Appendix*, Fig. S3*B*, and Dataset S1). In addition, assay validity was confirmed by robust β-catenin binding at WREs within the *AXIN2* locus in both subtypes (Fig. 3 *B*, *Center*).

About half of the peaks (766) were shared between E and F, while 426 were E-only and 223 F-only, revealing a conspicuous divergence in the physical activity of nuclear β-catenin (Fig. 3 *B* and *C* and Dataset S1). Motif enrichment analysis revealed that all three groups of peaks (shared, E-only, F-only) were enriched for TCF/LEF consensus (Fig. 3*D*). In addition, in the E-only set we detected motifs of p53 and members of the DLX homeobox family (DLX1/3/5), while in F-only, consistent with a more differentiated phenotype, we found motifs of GATA and HNF4A, transcription factors associated with hepatocyte differentiation and metabolic regulation. These motifs match those identified in the scATAC-seq and indicate that β-catenin targeted genomic regions are not universally predetermined but change even across closely related cellular subtypes.

E and F shared peaks were predominantly located close to the TSS (28% within 5 kb of the TSS), while subtype-specific peak regions are more gene-distant (only 5% of E-only and 13% of F-only are within 5 kb of the TSS; Fig. 3*E*). This indicated that one primary differentiator in β-catenin’s activity is the engagement with distal regulatory regions, likely subtype-specific enhancers. Signal profiles of all Wnt peaks separated by promoter or enhancer classification are in *SI Appendix*, Fig. S4*A*. Intersecting CUT&RUN peaks with subtype-specific differential gene expression allowed us to define the biological processes directly driven by β-catenin (Fig. 3 *F* and *G* and Dataset S2). Shared targets like *ZNRF3*, *RNF43*, *NKD1*, and *DKK1* confirmed universal activation of Wnt signaling (Fig. 3 *F* and *H*). E-only targets included genes for aggressive growth and invasiveness (*PCDH7, NRG1, FNDC3A,* and *SDK1*), implicating β-catenin in the regulation of the enhanced metastatic potential and therapy resistance of the E subtype (Fig. 3 *F* and *H*). F-only targets included metabolic genes such as *PAH*, *FABP1,* and *POR,* emphasizing that β-catenin regulates hepatic differentiation and metabolic specialization of the F subtype (Fig. 3 *F* and *H*). KEGG pathway enrichment analysis, using all expressed genes as background, confirmed this pattern: Shared β-catenin targets were associated with cancer pathways and Wnt signaling, E-only targets were enriched for stem cell pluripotency genes, and F-only targets for metabolic pathways and growth hormone synthesis (Fig. 3*G*). These data demonstrate that β-catenin contributes to orchestrating differential gene expression programs even in related cellular subtypes within the same tumor.

### Chromatin Accessibility Alone does not Explain Differential β-Catenin Binding Patterns.

**Fig. 4.**
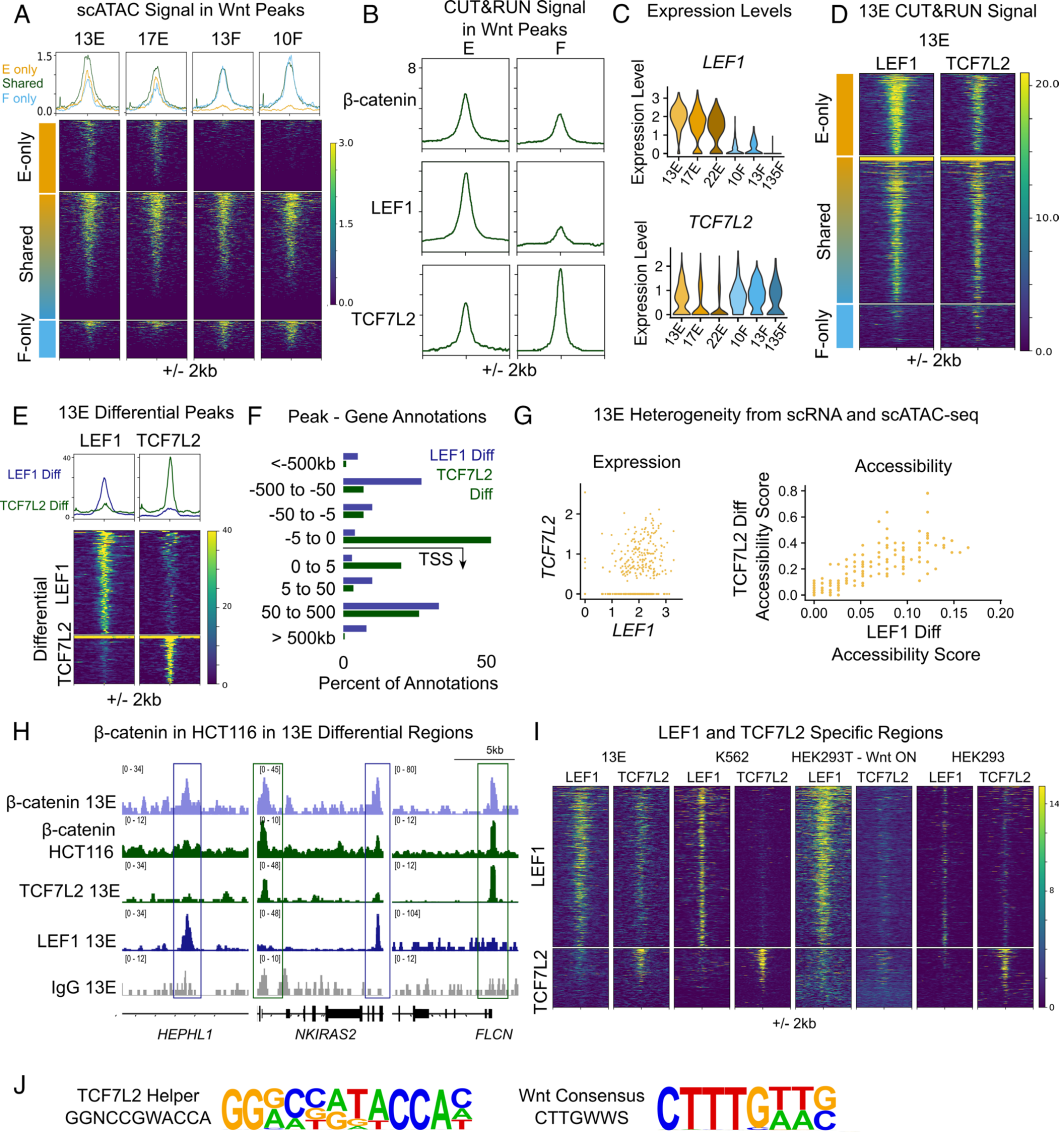
Differential activity of LEF1 and TCF7L2. (*A*) Chromatin accessibility scATAC-seq signal from 13E, 17E, 13F, and 10F plotted within Wnt peak subsets. E-only regions become inaccessible in F lines, while shared and F-only regions remain comparably accessible in both subtypes. (*B*) Signal profiles of β-catenin, LEF1, and TCF7L2 within Wnt peaks, showing the median profile. In E-lines (left, average of all three lines shown), β-catenin and LEF1 signal are higher than TCF7L2 signals, vs. in F-lines (*Right*), TCF7L2 signal is strongest. (*C*) *LEF1* and *TCF7L2* expression levels in the organoid lines according to scRNA-seq. Most E-lines predominantly express *LEF1*, and F-lines *TCF7L2*, except for 13E which expresses both. (*D*) Signal profiles of LEF1 and TCF7L2 CUT&RUN in 13E within Wnt peak regions. (*E*) Differentially occupied regions between LEF1 and TCF7L2 according to PePr (*P* < 0.00005, FC > 2) in 13E. (*F*) Peak-gene annotation via GREAT revealed that LEF1 differential regions are located distal to genes, vs. TCF7L2 differential regions are predominantly close to the TSS. (*G*) *Left*: scatter plot showing *LEF1* vs. *TCF7L2* expression, revealing that most cells express either only *LEF1*, or both, and there are not subpopulations that express only *TCF7L2.* *Right*: scatter plot of average accessibility score within LEF1 differential regions vs. TCF7L2 differential regions. The correlation indicates these regions are typically equally accessible within single cells and do not represent subpopulations within the 13E line. (*H*) Genomic browser example views of LEF1 and TCF7L2 differential regions for β-catenin CUT&RUN in HCT116 and data from 13E. (*I*) Heatmaps of LEF1 and TCF7L2 specific regions identified in at least 2 of 4 matched datasets for each protein. (*J*) Motif logos for the identified TCF7L2 helper site and the Wnt consensus motif.

A plausible explanation for the presence of E- and F-only β-catenin peaks could be differential chromatin accessibility in the two subtypes. We explored the scATAC-seq data focusing on Wnt peaks as defined in Fig. 3. In F organoids, the genomic regions corresponding to E-only peaks were mostly, yet with some exceptions, characterized by low scATAC-seq signal, suggesting that the vast majority of the underlying regulatory elements are not available for regulation by β-catenin in this context (Fig. 4*A* and *SI Appendix*, Fig. S4*A* for promoter/enhancer profiles). However, the E organoids displayed comparable open chromatin in the genomic regions corresponding to F-only peaks relative to F organoids (Fig. 4*A* and *SI Appendix*, Fig. S4*A*). Despite being accessible, the F-only regions are not actively engaged by β-catenin or TCF/LEF in E organoids. This indicated that chromatin accessibility, particularly in E organoids, and in a subset of loci in F organoids, do not fully account for the differential binding of β-catenin.

### Locus-Specific Partnership Between β-Catenin and TCF/LEF Transcription Factors.

We noticed that the CUT&RUN average signal of the two expressed TCF/LEF factors in PDOs, LEF1, and TCF7L2, depended on cellular subtype: In E cells, TCF7L2 signal was weaker than both LEF1 and β-catenin signals, while in the F lines this ratio is reversed and TCF7L2 was consistently the strongest (Figs. 3*C* and 4*B*). This pattern follows the switch in expression: E cells express higher *LEF1* which decreases in F cells in favor of *TCF7L2* (Fig. 4*C*), akin to hepatocyte differentiation in the liver (31). The LEF1-to-TCF7L2 expression switch is accompanied by a switch in genomic loci recognized and consequently bound by β-catenin (Fig. 3*C*). Therefore, it might be the different activities of LEF1 and TCF7L2 that confer locus-specific association to β-catenin. However, we could not exclude that additional context-specific cofactors drive the different genomic association of LEF1 and TCF7L2 in E vs. F, and these Wnt effectors would be redundant were they expressed in the same cells.

The decisive test for this came from the 13E organoid line, which robustly expresses both *LEF1* and *TCF7L2*. Accordingly, this line displays comparable LEF1 and TCF7L2 binding signal (Fig. 4*D*). We used PePr (32) to determine regions differentially bound by either LEF1 or TCF7L2 within the 13E line (*P* < 5 × 10^−5^, FC > 2, Dataset S3) and identified 131 regions where β-catenin partners only with LEF1, and 61 regions where it partners only with TCF7L2 (Fig. 4*E*). LEF1-only regions were predominantly gene-distal, likely functioning as enhancers, whereas TCF7L2-only peaks were mainly near the TSS, suggesting that β-catenin/LEF1 and β-catenin/TCF7L2 complexes preferentially act on different regions of the genome (Fig. 4*F*). Of note, both the LEF1-only and TCF7L2-only peaks showed comparable enrichment of β-catenin CUT&RUN signal (*SI Appendix*, Fig. S5*A*), lending credibility to these peak sets, and indicating that both must be considered as genuine Wnt/β-catenin target genes. Finally, scRNA-seq data of 13E cells shows that while some cells express only *LEF1*, most coexpress *LEF1* and *TCF7L2* (Fig. 4 *G*, *Left*). Importantly, scATAC-seq data showed that there are no cells which have open chromatin in exclusively LEF1-only or TCF7L2-only peaks (Fig. 4 *G*, *Right*). These data collectively show that each β-catenin molecule is directed to specific loci predetermined by which TCF/LEF it is associated to.

### Universal Association of LEF1 and TCF7L2 on Different Wnt-Responsive Elements.

We explored multiple datasets from us and others to test whether the skewed binding pattern of β-catenin toward the same LEF1-only and TCF7L2-only differential regions was universal. The HCT116 colorectal cancer line, which also carries activating mutations in *CTNNB1*, is a good test case of primary regulation by TCF7L2 (33). In these cells, β-catenin consistently binds to the majority of TCF7L2-only regions (45 of 61, 74%), but only to a minority of LEF1-only peaks (29 of 131, 22%). This pattern was confirmed by signal heatmaps and inspection of several loci, showcasing a β-catenin profile in HCT116 that largely matches that of the β-catenin/TCF7L2 complex in 13E organoid (Fig. 4*H* and *SI Appendix*, Fig. S5*B*). It is important to point out that HCT116 cells do not express LEF1, and TCF7L2 appears incapable of fully recapitulating LEF1 binding profile, confirming the selective nature of their different modes of action and the unexpected, limited redundancy. We also found several matched datasets of other cell types expressing both LEF1 and TCF7L2, including our previous CUT&RUN data in CHIR99021-stimulated HEK293T cells (Wnt-ON) (30), and ChIP-seq datasets in HEK293 and K562 cells from ENCODE (34, 35). Inspection of β-catenin signal across the Wnt peaks seems to follow the pattern identified in hepatoblastoma across all the models considered. (*SI Appendix*, Fig. S5*C*).

To identify cell-type agnostic sets of LEF1-only and TCF7L2-only peaks, we overlapped the peaks between the four matched datasets (13E, HEK293T Wnt-ON, HEK293, K562) for LEF1 and TCF7L2, and considered those peaks present in at least two different models. This yielded 2220 LEF1 and 983 TCF7L2 peaks. Of these, only 328 (ca. 10% of total) were shared between the two factors. We pinpointed 964 LEF1-only peaks (never called as TCF7L2 peaks; 0 of 4 datasets), and 354 TCF7L2-only peaks (never called as LEF1 peaks; 0 of 4 datasets). Contrary to the expected large redundancy in DNA-binding capabilities, our data identified considerable regions that are only bound by one of the two Wnt regulators (Fig. 4*I* and Dataset S3).

### A Novel TCF7L2 Long-Helper Motif Directs Genome-Wide Binding Specificity.

Our analyses showed that neither expression pattern, nor chromatin accessibility could explain the differential binding of LEF1 and TCF7L2. We tested whether this could be due to an intrinsic difference in their DNA-binding capability. De novo motif analysis on the LEF1-only and TCF7L2-only peaks revealed that while LEF1-only peaks were enriched for the TCF/LEF consensus (match score 0.95), the TCF7L2-only binding sites were characterized by an uncharacterized 5’-GGNCCGWACCA-3’ consensus sequence with no convincing match (top ranked was that of the PRDM family with a low score of 0.68) (Fig. 4*J*). This motif was almost identical to the top motif identified in the 13E TCF7L2 differential peaks (*SI Appendix*, Fig. S5*D*), and it contains, but it is not limited to, the RGGC helper motif bound by the C-clamp domain, present in TCF7 and TCF7L2 (23, 36–38). This motif is longer than and does not fully match the 5’-GCCGCCR-3’ helper site for TCF7 previously identified via genome-wide analysis in DLD1 cells by the Waterman lab (39).

We searched across the LEF1-only and TCF7L2-only peaks for the classical TCF/LEF motif: 5´-CTTTGWWS-3´, the short-helper: 5´-RCCG-3´, and the new TCF7L2 long-helper: 5´-GGRCCRTACCAC-3´. We found that the LEF1-only peaks are enriched for multiple TCF/LEF motifs with the same peak (obs/exp 1.55, logP −18.3), but not for co-occurrence of the TCF/LEF motif with either of the two helper sequences (obs/exp 0.94 and 0.74 for short helper, and TCF7L2 helper, respectively) and can be thought of as classical WREs. The TCF7L2-only peaks, on the other hand, were only mildly associated with repeats of the classic TCF/LEF motif (obs/exp 1.12, logP −2.58) and were also not characterized by co-occurrences of the classic motif with either helper sequence (obs/exp 1.0, 0.57, respectively). Instead, they were enriched for co-occurrence of multiple TCF7L2 long-helper sequences (obs/exp 1.68, logP −2.68), but not for co-occurrence of the short-helper (obs/exp 0.96), despite being contained within the TCF7L2 long-helper. Annotation of classical WRE motifs, short helpers, and TCF7L2 longer helper motifs in the original organoid Wnt peaks are available in Dataset S4. This indicated that the TCF7L2 long-helper motif is a functional unit that allows differentiation of binding sites specific for TCF7L2 from LEF1. Whether the long-helper might differentiate TCF7L2 from TCF7 that primarily uses the short-helper remains to be determined. These results unearth a long-helper recognition sequence for TCF7L2, which, even in the absence of a classical TCF/LEF motif, may facilitate DNA binding and Wnt target gene regulation. It is possible that TCF7L2 binds to this sequence directly, as is implied by the inclusion of the characterized C-clamp helper sequence, or that it relies on cell-type specific cofactors to associate to these DNA regions.

### TCF7L2 Helper Motif Mediates a Hepatic-Lineage Gene Program.

**Fig. 5.**
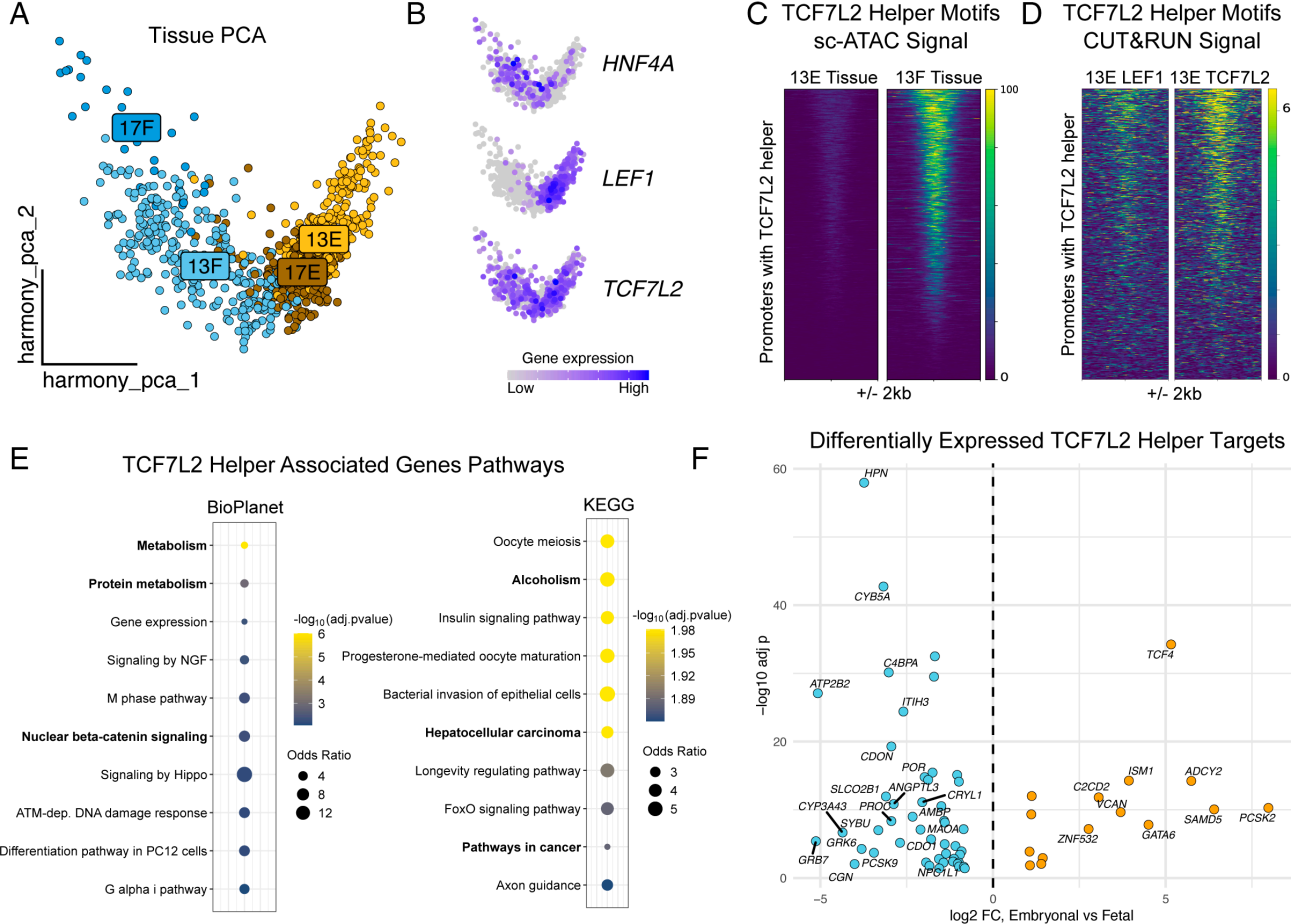
TCF7L2 directs a hepatic lineage gene program. (*A*) Integrated PCA plot of tumor cells from patient tissues. (*B*) Gene expression of *HNF4A*, *LEF1,* and *TCF7L2* plotted on top of the integrated PCA plot. (*C*) scATAC signal of 13E and 13F tissue cells within promoter regions containing TCF7L2 helper motifs, showing higher accessibility in 13F. (*D*) CUT&RUN signal for LEF1 and TCF7L2 from 13E organoids over promoter regions containing TCF7L2 helper motifs, showing higher signal in TCF7L2 compared with LEF1. (*E*) Pathway enrichment analysis for genes associated with TCF7L2 helper motifs. Both BioPlanet (*Left*) and KEGG (*Right*) databases result in enriched terms associated with the more differentiated hepatic phenotype. (*F*) Volcano plot showing differentially expressed TCF7L2 helper motif-associated genes within the tissue cells.

To investigate the gene expression programs potentially regulated by TCF7L2, we performed combined scRNA/ATAC-seq on tumor tissues of patients 13 and 17 (from which organoids 13E/F and 17E were derived, respectively). After filtering low quality cells and selecting for tumor cells, we identified clusters corresponding to E and F subtypes in both samples. Data were integrated using Harmony (40) and PCA revealed a separation of the E and F subtypes along the first principal component (Fig. 5*A*). The two subtypes in patient tumors expressed many of the same markers observed in our organoid dataset (*SI Appendix*, Fig. S6 *A* and *B*). Notably, this included a clear enrichment of expression of *LEF1* in the E subtype, *HNF4A* in the F subtype, and *TCF7L2* in both (Fig. 5*B*).

Next, we searched the open chromatin regions within the tissue for occurrences of the TCF7L2 long-helper motif. Of 2,752 motif instances, 844 (31%) were located within 5 kb of a TSS (Dataset S5). These regions were more accessible in F vs. E cells within the tumors (Fig. 5*C*). We plotted the CUT&RUN signal from the organoids within these TSS-adjacent regions and confirmed that they contain strong TCF7L2 signal enrichment and comparably weak LEF1 signal, validating the usage of this motif to identify differential targets of these two factors (Fig. 5*D*). When we explored the genes associated with these motifs, the highest enriched KEGG and BioPlanet pathways included hits like hepatocellular carcinoma (adj. *P* 0.01, odds ratio 3.67) and metabolism (adj. *P* 9.7 × 10^−7^, odds ratio 2.35), terms associated with a more differentiated F phenotype, and interestingly even nuclear β-catenin signaling (adj. *P* 0.005, odds ratio 6.32) (Fig. 5*E*). TCF7L2 helper motif-associated genes differentially expressed in F vs. E cells included key hepatic targets, such as *HPN*, *CYP3A43*, *PCSK9,* and *POR* (Fig. 5*F*). Our data suggest that DNA sequence recognition by different TCF/LEF family members is a primary mechanism for the divergent Wnt/β-catenin nuclear activity across different cell types, driving phenotypic differences.

## Discussion

One fundamental unresolved question in developmental biology is how signaling pathways, typically represented as linear series of events culminating in a pathway-specific transcriptional complex, can regulate gene expression in a tissue- and context-specific manner. The Wnt/β-catenin pathway exemplifies this complexity, and our inability to define its cell-type specific mechanisms hinders the identification of inhibitors that could target specific Wnt-driven cancers, without perturbing homeostatic Wnt signaling.

Here we leverage hepatoblastoma, a developmental liver cancer, as a unique model to study Wnt/β-catenin regulation given the presence of two distinct embryonal (E) and fetal (F) phenotypes within a tumor, despite sharing the common causative activating mutations in *CTNNB1*. The patient-derived organoid lines we employ preserve the E and F phenotypes and their defining transcriptional features. Single-cell transcriptomics combined with chromatin accessibility profiling provided reference datasets that reveal regulated gene expression modules, in which lineage-specific transcription factors drive the two distinct cell fates. To uncover the direct causative role of β-catenin in these genetic programs, we mapped binding sites of β-catenin and the related TCF/LEF factors with CUT&RUN, across all the models. This revealed that β-catenin exhibits subtype-specific activities aligned with their divergent fates, which is not explained by *CTNNB1* mutational status alone.

Our key finding, that the E and F subtypes are characterized, and perhaps defined, by a switch in *LEF1* to *TCF7L2* expression, has important implications. These two factors, while being largely redundant, have also been hinted to possess different functions. LEF1 acts as a transcriptional activator within progenitor-like contexts and was often associated with promoting epithelial-to-mesenchymal transition (EMT), stemness, and invasiveness in cancer (25). In contrast, TCF7L2 was shown to mediate differentiation, notably maintaining proliferation and metabolic function in tissues such as the intestinal epithelium and liver (22).

Our findings reveal a clear distinction between these two transcription factors, showing that they direct β-catenin to regulatory elements in a locus-specific manner. This difference is caused by recognition of a distinct DNA motif, likely bound by TCF7L2, and different from the classic WREs with which LEF1 mostly associates. TCF7L2 is one of the two TCF/LEF, in vertebrates, to possess the additional C-clamp domain that anchors it to WRE via an additional helper motif. Our identification not only sets TCF7L2 apart from the previous motif defined for TCF7 (39) but points to a TCF7L2 regulation of target genes that seems largely independent of the presence of classic WREs. This is surprising, as it expands the definition of Wnt target genes and shows that TCF/LEF might be more different than previously thought in their DNA binding capability. We find it surprising that it is the regulatory syntax of DNA elements, in addition to the accessibility of the sites, and the association with tissue-specific regulators, that defines subtype-specific Wnt/β-catenin targets. This is determined by the choice of TCF/LEF partner, a family of proteins historically viewed as largely redundant and distinguished mainly by tissue-specific expression patterns. Here, we found that even when both LEF1 and TCF7L2 are simultaneously present in individual cells, they can recruit β-catenin to their own distinct regulatory elements. In addition, we showed that this differential motif usage contributes to the establishment of distinct cellular and tumor phenotypes, even in related cell types that coexist within the same tumor.

Our data also shed light on the relationship between chromatin accessibility and β-catenin activity. E and F organoids express distinct Wnt target programs despite similar promoter accessibility at these genes, while the main difference in β-catenin binding was instead at distal regulatory elements. This is consistent with earlier findings that gene regulation is predominantly driven by enhancers rather than promoters during development (41, 42). Furthermore, our observation that F-specific Wnt peaks remain accessible yet unbound in E organoids fits with findings that chromatin accessibility is necessary but not sufficient for transcription factor binding and does not always predict gene expression (43–46). The choice of β-catenin’s binding partner thus represents a regulatory layer operating beyond chromatin state and may help explain similar observations of context-dependent DNA binding across other signaling pathways and cell types.

An important aspect that remains to be determined is the cooperation between the LEF1/β-catenin and TCF7L2/β-catenin complexes with lineage-specific transcriptional regulators. Our analysis suggests that such interaction might be contributing to their activity. The LEF1/β-catenin and TCF7L2/β-catenin complexes, in fact, drive expression of lineage-specific genes by binding to regulatory regions that carry motifs for lineage-determining transcription factors. A prominent example is HNF4A, a master regulator of hepatic differentiation, whose motifs are enriched in the F subtype, which is characterized by a more differentiated phenotype closer to adult hepatocytes. While several studies identified an interplay between β-catenin and HNF4A, a closer inspection into their shared genomic action and dependency is much needed to reveal new regulatory logic underlying Wnt-driven differentiation programs.

## Methods

### Organoid Culture.

Six organoid lines (embryonal 13E, 17E, and 22E and fetal 10F, 13F and 135F) were profiled and cultured as in Kluiver et al. (20) Organoids were maintained in liver tumor medium (Advanced DMEM/F-12 plus B27, N2, N-acetyl-l-cysteine, gastrin, EGF, 10% RSPO1 CM, HGF, nicotinamide, A83-01, forskolin, Y-27632 and next-generation Wnt surrogate) as described previously.

### Organoid Single-Cell Data Analysis.

Organoid scRNA-seq and scRNA/ATAC-seq datasets from Kluiver and Lu et al. were reanalyzed in R 4.4.0, and only data from the six models used in this study were retained. Analysis was performed using Seurat (47–49) (v5.1.0). For the scRNA-seq dataset, cells with 2 to 20% mitochondrial RNA were retained; the top and bottom 10% of nFeature_RNA per line were excluded, and all six lines were downsampled to 300 cells per line. Data were processed using SCTransform; where mitochondrial, cell-cycle, and cell-cycle correlating genes were removed from the variable features set as in Kluiver et al. (20) Dimensionality reduction used PCA and UMAP (5 dims). Differential expression between embryonal and fetal groups applied FindMarkers (min.pct = 0.5). The RNA/ATAC multiome analysis employed Seurat and Signac (50) (v1.14.0). The top and bottom 10% of nFeature_RNA and nFeature_ATAC per patient were removed, as well as percent.mt > 1.5 %, TSS.enrichment < 4 and nucleosome_signal > 2, and down-sampled to 130 nuclei per line. RNA layers followed the scRNA pipeline. Peaks were called using MACS3 (51) and further processed using TF-IDF, FindTopFeatures, SVD, and LSI, with standard Signac functions. Modalities were integrated with weighted-nearest-neighbor UMAP (FindMultiModalNeighbors). JASPAR 2024 motifs were added and chromatin deviations calculated with chromVAR 1.26.0.

### CUT&RUN LoV-U Experiments.

CUT&RUN Low Volume Urea, library preparation, and sequencing were performed as described in Zambanini et al. (30) with the following modifications. Organoids were harvested, removed from BME with dispase, dissociated to single cells with TrypLE, washed, and pelleted for 5 min at 800 g. Cells were resuspended in 2 mL nuclear extraction (NE) buffer [for 20 mL: 400 µL HEPES-KOH (1 M) pH 8.2, 200 µL KCl (1 M), 5 µL spermidine (2 M), 10 µL IGEPAL 100%, 8 mL glycerol 50%, 20 µL PMSF (1,000×), ddH2O to 20 mL], pelleted again, and washed a total of three times. Nuclei were finally resuspended in 40 µL NE buffer per sample. Nuclei pellets were frozen in an isopropyl chamber and stored at −80 °C until processed. CUT&RUN wash buffers were supplemented with 0.025% digitonin and 0.05% BSA. Antibodies used included anti-β-catenin (antibodies-online, ABIN2855042), anti-LEF1 (antibodies-online, ABIN1680678), anti-TCF7L2 (Cell Signalling Technologies, #2569S), and rabbit IgG isotype control (Invitrogen, #100500C). After pAG-MNase digestion, the reaction was stopped with 3 uL of EDTA/EGTA 250 mM mix, and fragments were released by the addition of 2 uL 5 M NaCl followed by heating to 37 degrees for 30 min. The supernatant was saved, and the beads resuspended in 50 uL 1× Urea buffer for the second release step. After 30 min incubation at RT, the urea release was combined with the initial supernatant. Samples were subjected to proteinase K digestion and SDS treatment before being purified using phenol chloroform isoamyl alcohol followed by ethanol precipitation. Samples were sequenced to a depth of 5 to 15 million reads on a NextSeq 550 with 36 bp pair-end reads.

### CUT&RUN Data Analysis.

Processing of CUT&RUN reads was performed as described by us previously (52). Reads were trimmed with bbmap bbduk (53) (version 38.18) to remove adapters and poly (AT), (G) and (C) repeat sequences. Reads were aligned to the hg38 genome with bowtie2 (54) (version 2.4.5) with the options –local –very-sensitive-local –no-unal –no-mixed -no-discordant –phred33 –dovetail -I 0 -X 500. Samtools (55) (version 1.11) suite was used to remove duplicate and incorrectly paired reads. Bedtools (56) (version 2.30.0) was used to remove reads mapped to the CUT&RUN hg38 suspect list (57) from bam files. Bigwigs were created for visualization using deepTools (58) (version 3.5.1-0) with the options −e and −RPCG. After quality control of individual datasets, biological duplicates were merged using samtools. Peaks were called on merged datasets using GoPeaks (59) with the options −r 8 and the default threshold of adj. *P* < 0.05, against the corresponding negative control. Wnt peaks were defined for each organoid subtype by combining peaks from β-catenin, LEF1, and TCF7L2 for each organoid line, and then determining regions called in at least 2 of 3 lines. PePr (32) was used to define differential peaks, inputting the individual biological replicates and negative controls, with the settings *P* < 0.00005 (default) and subsetting for a calculated FC > 2. Final peak sets were generated after subtracting peaks that consisted of >50% soft-masked or marked repeat regions. For figure visualization, bigwigs of all three organoid lines per subtype were averaged using deepTools bigwigAverage. Heatmaps and signal intensity plots were created with deepTools computeMatrix and plotHeatmap. Motif analysis was done using HOMER (60) (version 4.11) findMotifsGenome to find motifs in the hg38 genome using -size 200, and annotatePeaks for motif searching and identification. Peak set gene annotation was done using GREAT (61) (version 4.0.4) with default parameters. Enrichr (62) was used for pathway analysis with KEGG and/or Bioplanet pathways, using the entire set of expressed genes according to the scRNA-seq as a background set. Public data for β-catenin in HCT116 (33), and LEF1 and TCF7L2 in HEK293T (30), K562 (ENCSR343ELW and ENCSR888XZK), and HEK293 (ENCSR240XWM and ENCSR000EUY) were downloaded and compared using the bigwig and peak files.

### Patient Tissue Samples.

This research project complies with all relevant ethical regulations. This project was approved by the Princess Máxima Center Biobank and Data Access Committee (PMCLAB2020-107). Fresh tumor specimens were obtained from surgical resections performed at the University Medical Center Groningen (UMCG) with written informed consent from patients and/or their legal guardians.

### Patient Tissue Single-Cell RNA/ATAC Sequencing.

Single-cell ATAC and gene expression samples and libraries were prepared using the 10× Genomics Chromium Next GEM Single Cell Multiome ATAC + Gene Expression workflow, following the manufacturer’s instructions (CG000375 Rev C and CG000338 Rev F). Cryopreserved tissue fragments were thawed, washed in ice-cold PBS with 5% FBS, and minced to ~1 to 2 mm2 if needed. Nuclei were released by 5 min NP-40–based lysis with dounce homogenization on ice, filtered through 70 μm cell strainers (Greiner Bio-One EASYstrainer, Thermo Fisher), and pelleted at 4 °C. Pellets were resuspended in PBS with 2% BSA and Protector RNase inhibitor (Sigma-Aldrich). Intact nuclei were enriched by sorting on a Sony SH800S cell sorter with 100 μm nozzle for 7-AAD (Invitrogen) positive singlets and collected into BSA-coated tubes containing Protector RNase inhibitor. Nuclei were permeabilized, washed, counted on an automated counter (Countess II cell counter, Thermo Fisher) or hemocytometer, and adjusted to ~600 to 700 nuclei/μL for loading on a Chromium Controller. Libraries were sequenced on a NovaSeq6000 System (Illumina) with sequencing settings recommended by 10× Genomics and processed using Cell Ranger ARC (v2.0.0).

### Patient Tissue Single-Cell RNA/ATAC Analysis.

We imported 10× Genomics Single Cell Multiome matrices with Read10X_h5, created a Seurat object from RNA counts, and added ATAC data with Signac using hg38 and EnsDb.Hsapiens.v8637–40. ATAC peaks were called with MACS3 (51), peaks overlapping the hg38 unified blacklist were removed, and a peaks assay was built from fragments. SNP-based demultiplexing was performed using Python packages cellsnp lite (v1.2.2) and Vireo (v0.2.3). Genotyping references of the donors were obtained from bulk RNA-sequencing data of the tumor biopsy samples generated in the diagnostic setting. Entries labeled “doublet” or “unassigned” were excluded. For each sample, we trimmed the bottom and top 10 percent of cells by nFeature_RNA and nFeature_ATAC, then applied global filters of mitochondrial RNA fraction below 5 percent, TSS enrichment above 3, and nucleosome signal below 3. ATAC data were processed with TF-IDF, feature selection, and SVD, then corrected across reactions with Harmony (40) in LSI space. RNA data were split by reaction and normalized with SCTransform; features correlated with cell cycle or mitochondrial signal were removed using a custom correlator together with S and G2M gene sets, after which PCA was computed. Layers were integrated with IntegrateLayers (method = HarmonyIntegration) to obtain a shared Harmony-corrected PCA space (“harmony.pca”). Embryonal (E) and fetal (F) tumor populations for both patients were defined by analyzing each tumor separately after SCTransform and PCA at low clustering resolution, then assigning states based on canonical markers, with *LEF1* high indicating embryonal and *ALB* and *HNF4A* high indicating fetal. These assignments yielded four groups, 13E, 13F, 17E, and 17F, which were annotated to the full object. We downsampled the object to 300 cells per group, reran Harmony on LSI and PCA with 10 dimensions, and visualized *HNF4A*, *LEF1*, and *TCF7L2* on the Harmony PCA. Differential gene expression between embryonal and fetal populations was performed in Seurat using FindAllMarkers on the RNA assay.

### Western Blot.

Organoids were released from their matrix using dispase and lysed in RIPA Lysis Buffer System (Santa Cruz). After quantification using the BCA protein Assay Kit (Thermo Fisher), protein samples were separated by sodium dodecyl sulfate-polyacrylamide gel electrophoresis (SDS-PAGE). Proteins were then transferred onto polyvinylidene difluoride (PVDF) membranes (Millipore) using a semidry method (Trans-blot Turbo transfer system). Membranes were blocked in 5% BSA (Sigma-Aldrich) for 1 h at room temperature and incubated with anti-TCF7L2 (1:1,000, Cell Signaling Technologies, #2569), anti-LEF1 (1:1,000, Cell Signaling Technology, #2230), and GAPDH (1:2,000, Cell Signaling Technology, #2118) antibodies, followed by incubation with IRDye 800CW goat anti-rabbit IgG secondary antibody (1:5,000, LI-COR) and IRDye 680RD goat anti-mouse IgG secondary antibody (1:10,000, LI-COR). Immunoreactive proteins were subsequently visualized using the Bio-Rad ChemiDoc Imaging System.

### RNA Interference of LEF1/TCF7L2.

13E and 13F organoids were sequentially dissociated with dispase and TrypLE-Express to obtain single-cell suspensions. Tissue culture (TC)-treated plates were coated with 1% Matrigel diluted in organoid culture medium. Cells were then seeded and cultured in organoid culture medium supplemented with 2% FBS. For RNA interference, cells at 30 to 50% confluence were transfected with negative control siRNA (siCtrl; SIC001, Sigma-Aldrich), siRNA targeting LEF1 (siLEF1; EHU002271, Sigma-Aldrich), siRNA targeting TCF7L2 (siTCF7L2; s13880, Thermo Fisher Scientific), or a combination of siRNAs targeting LEF1 and TCF7L2, using Lipofectamine RNAiMAX (Thermo Fisher Scientific) according to the manufacturer’s instructions. Gene expression was evaluated 72 h posttransfection. For proliferation assays, cells were retransfected with siRNAs after 72 h and further incubated for an additional 96 h before performing Cell Counting Kit-8 assays.

### Real Time Quantitative PCR.

RNA was extracted with the RNeasy Mini Kit (Qiagen) and total RNA was reverse transcribed into cDNA employing GoScript™ Reverse Transcriptase (Promega) following the supplier’s protocol. RT-PCR was conducted in a 20 µL reaction mixture, which contained 50 ng of cDNA, 0.5 µM of forward and reverse primers and GoTaq® qPCR Master Mix (Promega) using a CFX384 Real-Time PCR Detection System (Bio-Rad). All primers were purchased from Integrated DNA Technologies (IDT), with sequences shown in *SI Appendix*, Table S1.

### Cell Viability Assays.

Cell viability assays were performed using Cell Counting Kit-8 (CCK-8). Transfected cells were cultured in a 48-well or 96-well plate. At the indicated time points, 10 μL of CCK-8 solution (HY-K0301, MedChemExpress) was added to each well, followed by incubation at 37 °C for 1 h. Absorbance at 450 nm was measured using CLARIOstar Plus microplate reader (BMG Labtech).

### TCF7L2 Isoform Identification.

cDNA templates were obtained by reverse transcription of total RNA isolated from 13E and 13F patient-derived organoids using GoScript™ Reverse Transcriptase (Promega). The target sites for all primers in the TCF7L2 genome are shown in *SI Appendix*, Fig. S1*E*. To identify TCF7L2 isoforms, a two-stage PCR strategy was employed. In the first stage, the 12 to 17 amplicon was delimited by 12_F/17_R primer pair, spanning the major alternatively spliced exons containing the C-clamp region. In the second seminested PCR stage, the gel-purified first-round PCR product (12 to 17 amplicon), recovered using the NucleoSpin Gel and PCR Clean-up kit (740609, Macherey-Nagel), was used as the DNA template. Specific exon regions were then defined using the primer pairs 13_F/17_R, 14_F/17_R, 15_F/17_R, and 16_F/17_R. In both PCR stages, amplification was performed using the designed primers (*SI Appendix*, Table S1) and Q5® High-Fidelity 2× Master Mix (New England Biolabs). The thermal cycling conditions were as follows: initial denaturation at 98 °C for 30 s; 35 cycles of denaturation at 98 °C for 10 s, annealing at 52 °C for 30 s, and extension at 72 °C for 15 s; final extension at 72 °C for 2 min. The specificity of PCR product was verified by electrophoresis on 3% agarose gels at a constant voltage of 120 V for 75 min.

## Supplementary Material

Appendix 01 (PDF)

Dataset S01 (XLSX)

Dataset S02 (XLSX)

Dataset S03 (XLSX)

Dataset S04 (XLSX)

Dataset S05 (XLSX)

## Data Availability

All data have been deposited in EGA (EGAS50000001229) (63).
