# Supplementary material for: Differential Wnt/β-catenin signaling via TCF7L2/LEF1 binding specificity shapes cellular and tumor phenotypes
Source: Proc Natl Acad Sci U S A. 2026 Jun 10;123(24):e2528450123. doi: 10.1073/pnas.2528450123 (PMC13273282; doi:10.1073/pnas.2528450123)
Supplement: Supplementary file 1 — Appendix 01 (PDF) [file pnas.2528450123.sapp.pdf]

SI Appendix

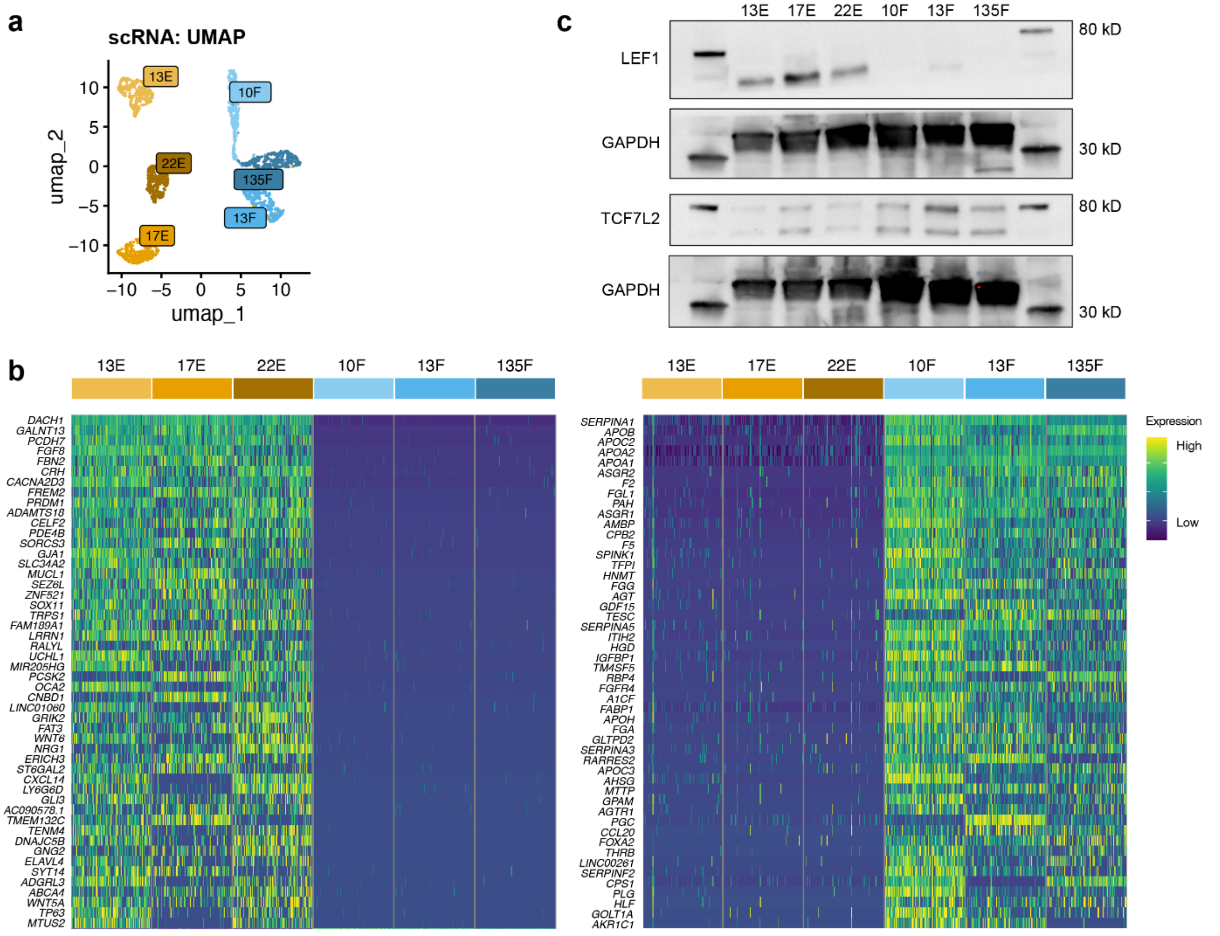

Figure S1. Organoid scRNA-seq analyses. a. scRNA-seq UMAP of E and F tumor organoid models. b. Heatmap showing the top 50 differentially expressed genes in E and F organoids. c. Western blots showing LEF1 and TCF7L2 in all organoid models with their respective GAPDH loading control.

d

TCF7L2 isoforms schematic

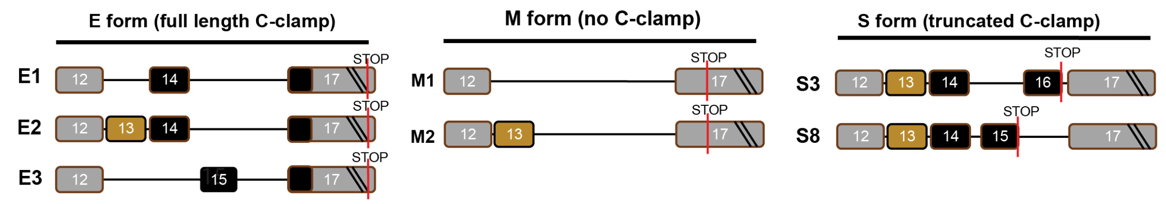

e

TCF7L2 isoform identification PCR primer strategy

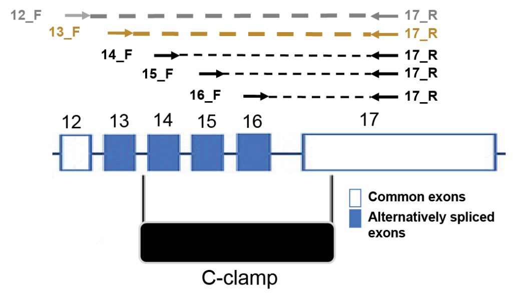

Amplicon lengths

Iso	AS exon code	13-17 bp	14-17 bp	15-17 bp	16-17 bp	12-17 total bp
E1	12-14-17	-	125	-	-	204
E2	12-13-14-17	201	125	-	-	255
E3	12-15-17	-	-	156	-	204
M1	12-17	-	-	-	-	131
M2	12-13-17	128	-	-	-	182
S3	12-13-14-16-17	226	150	-	117	280
S8	12-13-14-15-17	274	198	156	-	328

TCF7L2 isoform identification PCR gels

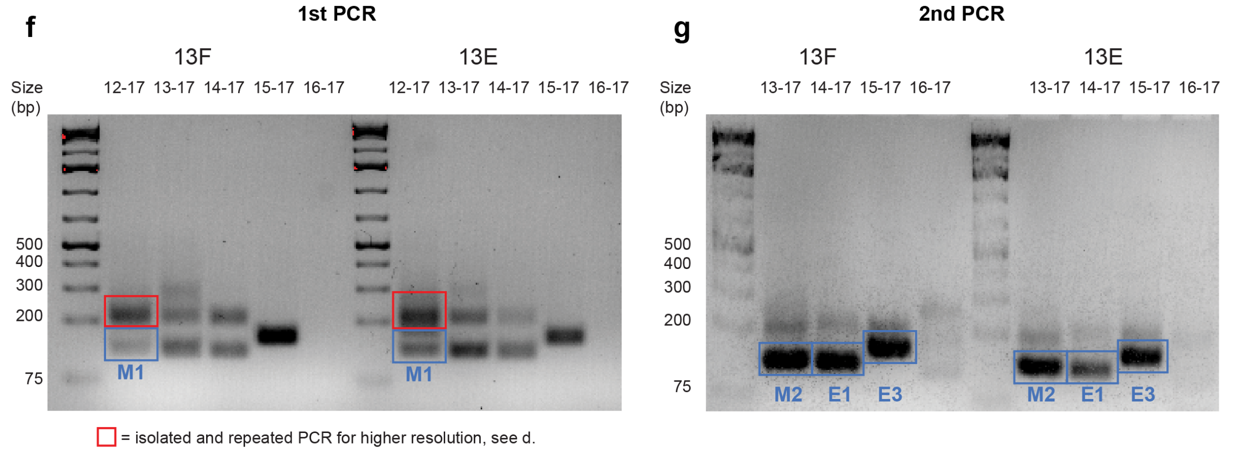

d. Schematic overview of different TCF7L2 isoforms. e. Schematic PCR primer locations to assay which isoforms are expressed in the organoids (left). Table showing potential amplicon lengths for each PCR and isoform combination (right). f. Agarose gel electrophoresis of PCR amplicons from the noted primer pairs, in 13E and 13F. We could identify the M1 isoform (131 bp). As some of the signal was likely off-target amplification, we isolated and purified the band amplified by primers 12-17 for a second PCR. g. Agarose gel electrophoresis of PCR amplicons from this second PCR, with the isolated amplicons (12-17) from the first PCR as input. We could identify M2, E1 and E3 isoforms.

h

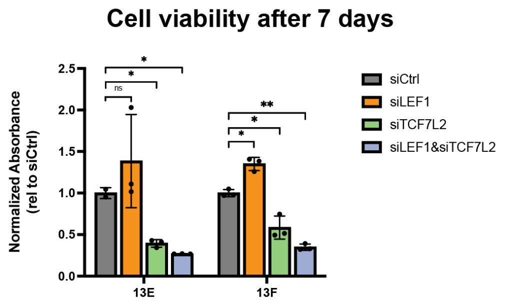

i

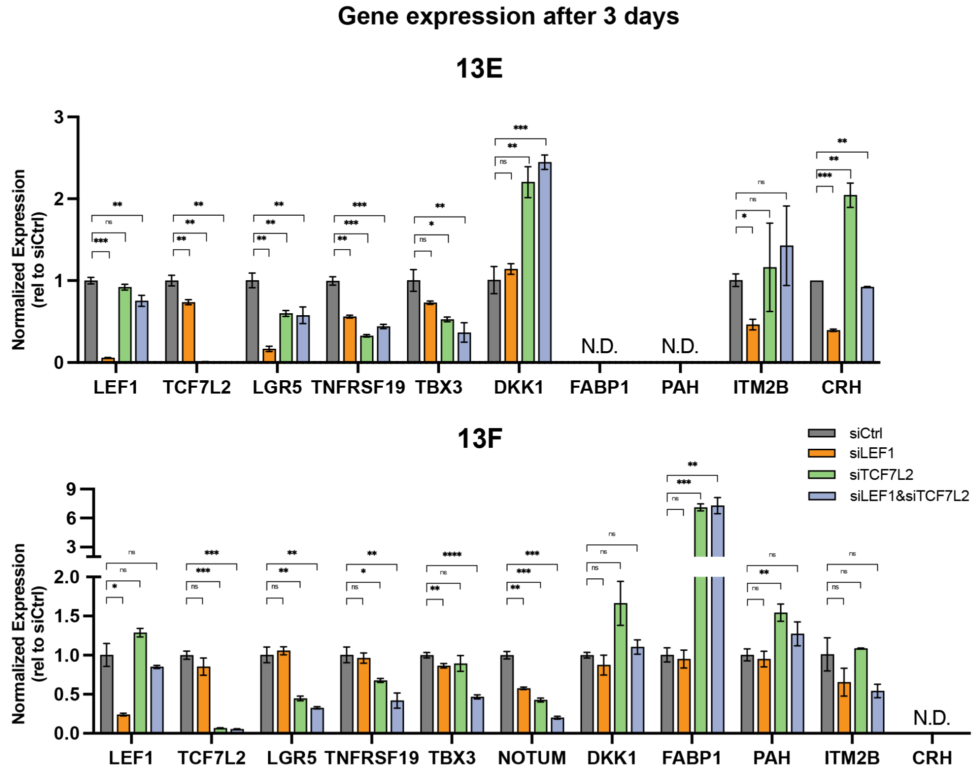

h. Bar plots showing viability of 13E and 13F cells after 7 days of treatment with 5.5 nM siCtrl (NC), 5.5 nM siLEF1, 5.5 nM siTCF7L2 or a combination of 5.5 nM siLEF1 and 5.5 nM siTCF7L2, assessed using Cell Counting Kit-8 assays (n = 3 per group). i. Bar plots showing gene expression analysis of 13E and 13F cells following 3-day treatment with 5.5 nM siCtrl (NC), siLEF1, siTCF7L2 or a combination of siLEF1 and siTCF7L2 by qPCR. Data are represented as mean ± SD of three measurements. Statistical analyses were conducted using unpaired t-tests with GraphPad Prism 10. N.D.: not determined. Significance levels were defined as follows: ns = $p > 0.05$, * = $p \leq 0.05$, ** = $p \leq 0.01$, *** = $p \leq 0.001$, **** = $p \leq 0.0001$.

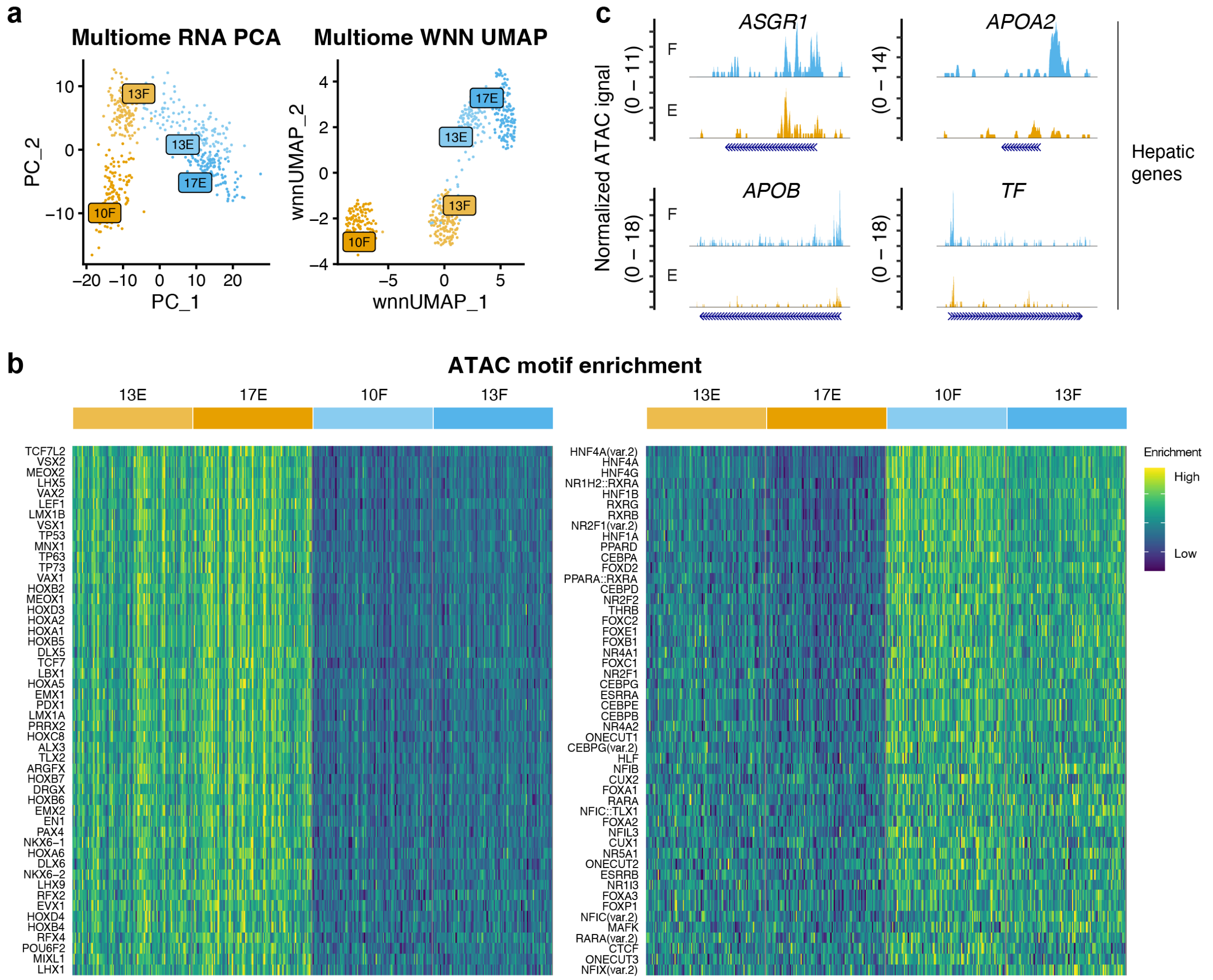

Figure S2. Chromatin accessibility analysis of patient-derived organoids (PDOs). a. scRNA/ATAC-seq PCA and Weighted Nearest Neighbor (WNN) UMAP of E and F tumor organoids. b. Heatmap showing differentially enriched motifs in E and F organoids. c. Coverage plots showing ATAC signal at promoter regions of selected hepatic targets split for E and F organoids.

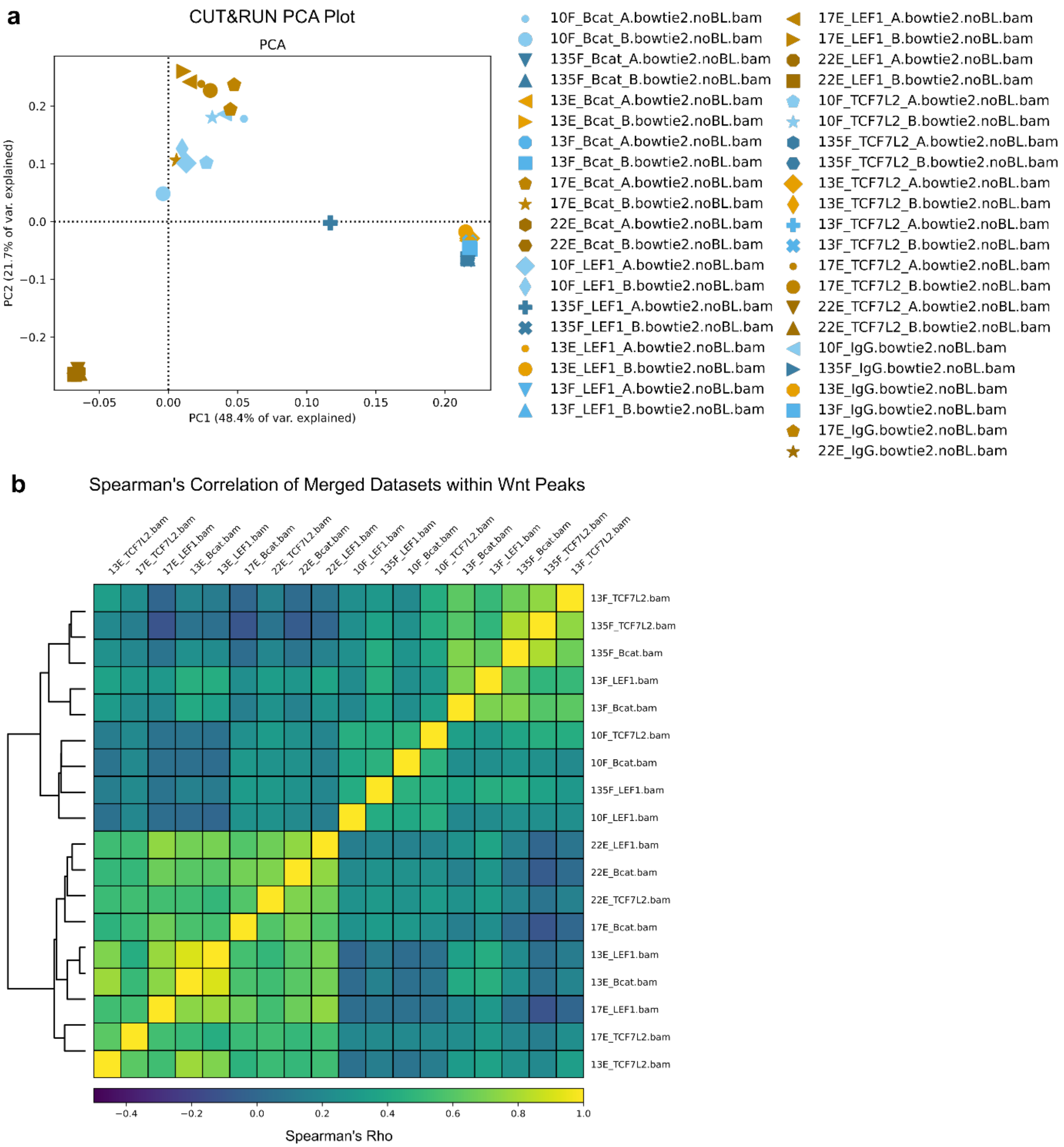

Figure S3. CUT&RUN LoV-U of PDOs. a. PCA plot after genome-wide binning of individual replicates, showing mainly separation based on genotype, due to karyotypic differences between the organoid lines. b. Spearman's correlation heatmap showing correlation of merged replicates within Wnt peaks. Two main unsupervised hierarchical clusters are formed, separating E and F lines.

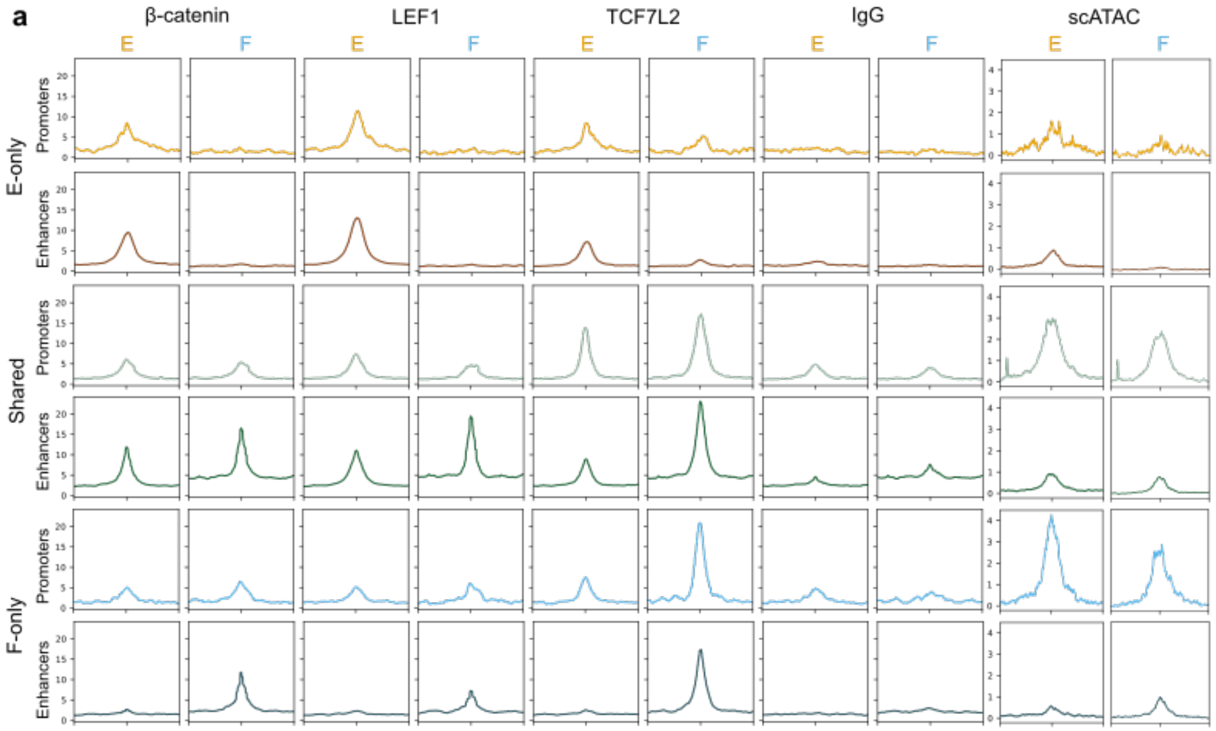

Figure S4. Signal Profiles of CUT&RUN LoV-U and scATAC-seq in promoter and enhancer Wnt peaks. a. Average signal profiles of CUT&RUN LoV-U datasets and scATAC-seq data within Wnt peaks, separated based on E-only, shared, and F-only status, and subclassified based on HOMER annotation for promoters, or putative enhancers (all other regions not annotated as promoters).

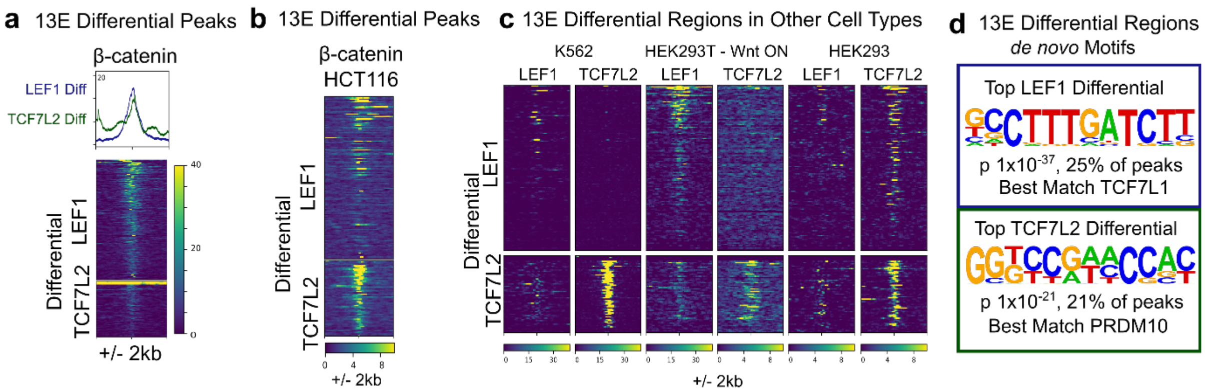

Figure S5. Differential LEF1 and TCF7L2 occupied loci in 13E. a. Heatmap showing β-catenin CUT&RUN signal within 13E differential regions, showing comparable average signal strength. b. Heatmap showing β-catenin CUT&RUN signal on 13E differential regions in *TCF7L2*-expressing HCT116 cells, showing higher occupancy in the TCF7L2 peaks. c. Heatmaps of LEF1 and TCF7L2 signal in published matched ChIP and CUT&RUN datasets of cells expressing both LEF1 and TCF7L2, on the 13E differential regions. d. Top *de novo* motifs identified in the LEF1 and TCF7L2 differential regions.

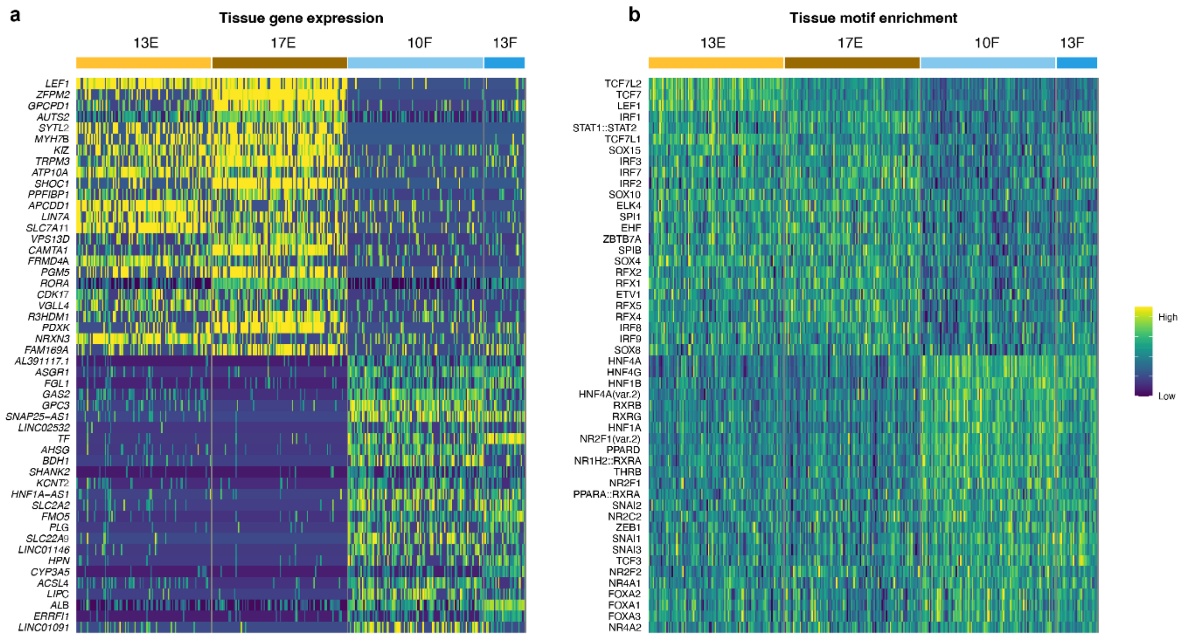

Figure S6. TCF7L2 directs a hepatic lineage gene program. a. Heatmap showing the top differentially expressed markers between the E and F subtypes in tissue samples from patients 13 and 17. b. Heatmap showing the top differentially enriched motifs in ATAC peaks between the E and F subtypes in patient tissue samples from patients 13 and 17.

Table S1: Primer sequences used in this manuscript.

qPCR primers:

Gene	Forward Sequence (5'-3')	Reverse Sequence (5'-3')
CRH	GGGAACCTCAACAAGAGCCC	AACACGCGGAAAAAGTTGGC
DKK1	ACAACTACCAGCCGTACCC	GAGACAGATTTGCACGCCT
FABP1	AAGACAGTGGTTCAGTTGGAAG	TGAGTTCGGTCACAGACTTGAT
GAPDH	CCACCTTTGACGCTGGG	CATACCAGGAAATGAGCTTGACA
ITM2B	TTGCCTCAGTCCTATCTGATTCA	TCTGCGTTGCAGTTTGTAAGT
LEF1	CCGAAGAGGAAGGCGATTTAGC	GGTCCCTTGTTGTAGAGGCC
LGR5	CTCCCAGGTCTGGTGTGTTG	GAGGTCTAGGTAGGAGGTGAAG
NOTUM	AACGCAAACATGGTCTTCATCC	GGCGTACTCGTTCTTCTCAGA
PAH	GCCTGCTCTGACAAACATCAT	TTATCTCGTGAAAGCTCATGGAC
TBX3	AGCCTGTTCCCTTACCCCTAC	GTTCAGAGCCCGAGTCCACT
TCF7L2	ATGCTTCCATGTCCAGGTTC	CACTCTGGGACGATTCCTGT
TNFRSF19	GGTGCATTCTGCAGCCAGTCTT	CAGGCATCTGAAAACTCGCCAC

TCF7L2 Isoform PCR primers:

Name	Sequence (5'-3')
12_F	AAGAAGAAGAAGAGGAAAAGGG
13_F	GAATGTTTCCTAAATCCTTGCC
14_F	TTGGCCTTGATCAACAGAATAA
15_F	GCAAATACTCCAAAGAAGTGTC
16_F	CTTGCAGTCTTTGAATTTGGAA
17_R	CGAATCTAGTAAGCTTCCATCT

SI Dataset Legends

Dataset S1: Chromosome coordinates of common, E-only and F-only Wnt peaks.

Dataset S2: Gene lists of peak-associated genes for common, E-only, and F-only Wnt peaks.

Dataset S3: Chromosome coordinates of differential LEF1 and TCF7L2 peaks from 13E organoids and from matched published datasets.

Dataset S4: Annotation of TCF/LEF, short helper, and TCF7L2 long-helper motifs within 200 bp and 500 bp Wnt peak regions.

Dataset S5: Chromosome coordinates and gene annotations for open chromatin promoter regions containing a TCF7L2 long-helper motif.
